# A Case Report of Idiopathic Omental Infarction in an Obese Child

**DOI:** 10.1155/2012/513634

**Published:** 2012-08-16

**Authors:** Tomoyuki Tsunoda, Tsuyoshi Sogo, Haruki Komatsu, Ayano Inui, Tomoo Fujisawa

**Affiliations:** Children's Center for Health and Development, Saiseikai Yokohamashi Tobu Hospital, 3-6-1 Shimosueyoshi, Tsurumi, Kanagawa, Yokohama 230-0012, Japan

## Abstract

Omental infarction (OI) is a rare cause of acute abdomen in children. A 9-year-old girl was presented with sudden-onset intermittent right lower quadrant abdominal pain and fever (37.9°C). Physical examination revealed abdominal tenderness in the right lower quadrant with localized rebound tenderness which resembled acute appendicitis. She was obese and her BMI was on the 99th percentile. Computed tomography (CT) revealed a 5 cm ill-defined heterogeneous fatty mass with hyperattenuating streaks just beneath the abdominal wall. She was diagnosed as OI and treated conservatively with reduced meals and antibiotics. Her symptom resolved gradually and she was discharged on day 7 without complications. OI should be considered as a differential diagnosis for acute right-sided abdominal pain, especially in obese children. Enhanced CT is useful for differentiating OI from other conditions presenting with acute abdomen.

## 1. Introduction

Omental infarction (OI) is a rare cause of acute abdomen in children. The symptoms of OI are similar to those of acute appendicitis and more than 90% of cases present with right-sided abdominal pain, making it indistinguishable without radiological examination [1, 2].

OI is classically categorized into primary and secondary cases. Secondary cases are related to surgery, hypercoagulopathy, or vasculitis. Primary cases are supposed to be caused by anomalous arterial supply to the omentum, kinking of veins associated with increased intraabdominal pressure, or vascular congestion after large meals [3]. One of the known risk factors for OI is obesity. We encountered a girl with primary OI whose body mass index (BMI) was in the 99th percentile and discussed the risk of obesity and the diagnostic usefulness of computed tomography (CT).

## 2. Case Presentation

**Figure 1:**
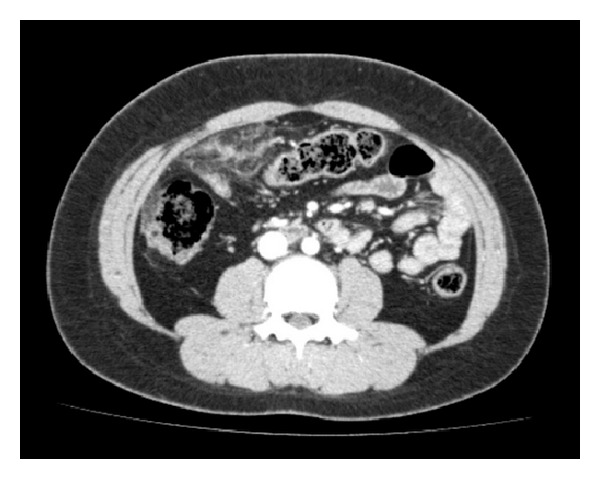
Enhanced CT reveals a 5 × 8 cm ill-defined heterogeneous fatty mass with hyperattenuating streaks just beneath the abdominal wall anterior to the transverse colon.

A 9-year-old girl was referred to our hospital with sudden-onset intermittent right lower quadrant abdominal pain and fever (37.9°C). Although her history had been uneventful until this episode, her BMI was in the 99th percentile. Despite abdominal pain and slight fever, she had a good appetite without gastrointestinal symptoms such as nausea or diarrhea. Physical examination revealed abdominal tenderness in the right lower quadrant with localized rebound tenderness. Blood examination showed findings of inflammation (white blood cell (WBC) count, 13,170/*μ*L with 80% neutrophils; C-reactive protein (CRP), 3.2 mg/dL) and coagulopathy (fibrin degenerative products (FDP), 14.5 *μ*g/mL; D-dimer, 3.8 *μ*g/mL). A 5 cm ill-defined heterogeneous fatty mass was demonstrated on plain CT and an area of high-attenuated fat with hyperattenuating streaks was revealed just beneath the abdominal wall on enhanced CT (Figure 1). The appendix was normal on CT. The characteristic CT findings with elevated FDP and D-dimer levels were suggestive of OI. We treated her conservatively with reduced meals and antibiotics. The WBC count and CRP level on day 3 of hospitalization were still elevated to 11,550/*μ*L and 11.4 mg/dL, respectively, although her symptoms were resolving. She was discharged on day 7 without complications.

## 3. Discussion

Although the first report on OI was published in 1896 by Bush [4], there are still less than 400 published reports about OI to date, including about 50 reports involving children. With the advent of imaging modalities, more than half of these reports have been published since 1990. Park et al. [1] collected 43 cases of OI over 10 years and reported that the male-to-female ratio was 2.58 : 1, the mean age was 31.7 years (range, 6–82 years), and 32.6% of the cases occurred in children aged under 15 years. In a previous report, the proportion of children among all patients with OI was 15% [5]. Obesity is a known risk factor for OI, and the increasing rate of child obesity may result in an increasing prevalence of OI in children [6, 7].

The greater omentum comprises right and left ligaments. The right ligament is supplied by branches of the gastroepiploic arteries. The right side predilection of OI is thought to be caused by the fact that the omentum is longer and more mobile on the right side. The greater omentum is transparent at birth and progressively accumulates fat, and the amount of fat corresponds to the BMI. Increased fat deposition may outstrip the blood supply to the developing omentum, causing relative ischemia, or the increased omental weight may lead to torsion [6]. A search of PubMed for English articles containing the terms “omental” and “infarction” since 2000 produced 12 articles that mentioned the relevance of OI to obesity in childhood cases. Among 91 children with OI, all of their weights were above the 50th percentile and at least 42 children (57%) weighed more than the 90th percentile for their age.

In most cases, the symptoms appear as acute right-sided abdominal pain resembling acute appendicitis. Moreover, 0.1–0.3% of children undergoing laparotomy for suspected appendicitis were finally diagnosed with OI [6]. However, unlike acute appendicitis, OI manifests no or slight gastrointestinal symptoms. In our case, the patient was also generally well with a good appetite, despite right abdominal pain. OI is thought to be a benign self-limited condition and the treatment only requires conservative management. Therefore, the exclusion of other mimicking diseases requiring surgery is important [1]. Plain CT demonstrates an ill-defined mass located between the abdominal wall and the transverse or ascending colon corresponding to a location in the greater omentum. Enhanced CT demonstrates a heterogeneous fatty mass with hyperattenuating streaks that is usually more than 5 cm in size [3]. Although ultrasound shows a hyperechoic cake-like soft tissue mass, it is less specific, can lead to misinterpretation, and is not useful in making a differential diagnosis [2, 3]. Obesity is a risk factor for OI but ultrasound is not a suitable examination for obese patients. Enhanced CT is a useful tool for identifying the characteristic signs of OI and is also useful for making a differential diagnosis. In our case, a typical heterogeneous fatty mass with hyperattenuating streaks was detected.

WBC counts and CRP levels are usually reported to be normal or slightly elevated [1, 6, 7]. However, in our case, the serum CRP level after hospitalization remained elevated despite improvements in the symptoms and the WBC count. This discrepancy was possibly caused by a relative delay of the CRP elevation. No previous reports have mentioned serial examinations of the WBC count and CRP level. However, considering that the symptoms are abrupt in onset, the patients tend to consult a physician within a short period of time and the CRP level may reach a peak a few days later.

In conclusion, OI should be considered as a differential diagnosis for acute right-sided abdominal pain, especially in obese children. Enhanced CT is useful for differentiating OI from other conditions presenting with acute abdomen.
